# Ventricular Extension of Intracerebral Hemorrhage during Intravenous Thrombolysis

**DOI:** 10.1159/000355144

**Published:** 2013-10-30

**Authors:** George W.J. Harston, Yee Kai Tee, Melanie Jones, Stephen Payne, George Pope, Fintan Sheerin, James Kennedy

**Affiliations:** ^a^Acute Stroke Programme and Radcliffe Department of Medicine, Oxford, UK; ^b^Acute Vascular Imaging Centre, Radcliffe Department of Medicine, Oxford, UK; ^c^Institute of Biomedical Engineering, Department of Engineering Science, University of Oxford, Oxford, UK; ^d^Oxford University Hospitals NHS Trust, Oxford, UK

## Case Report

A 69-year-old woman presented to the emergency department with sudden-onset left-sided weakness and sensory loss, neglect and right gaze preference. Acute ischemic stroke was diagnosed and the National Institutes of Health Stroke Scale score was assessed as 19. Her blood pressure was 179/81 mm Hg. Head computed tomography (CT) showed early ischemic change and the Alberta Stroke Program Early CT score was 8 without evidence of hemorrhage [1]. There was no contraindication to thrombolysis and alteplase was administered 95 min after the onset of her symptoms.

**Fig. 1:**
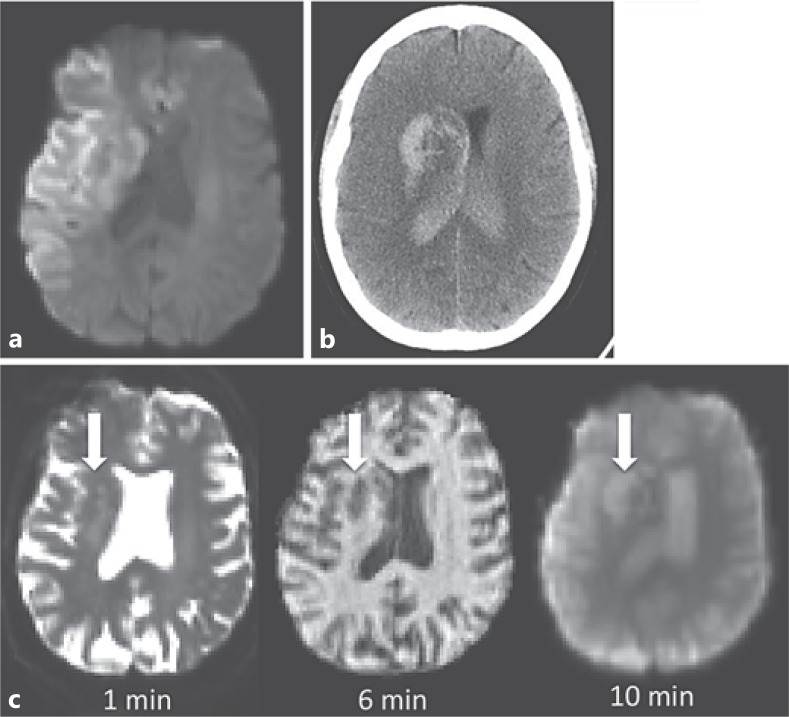
**a** Diffusion-weighted imaging 95 min after symptom onset demonstrating a large volume of tissue infarction within the right hemisphere. **b** Noncontrast CT scan at 180 min showing hemorrhage within the right basal ganglia and extension into both lateral ventricles. **c** Serial coregistered images during the MRI showing the appearance and growth of hematoma (arrows) over time from the beginning of the scan. Diffusion-weighted sequence: b = 0 s/mm^2^; T1-weighted image; arterial spin-labeled perfusion image, unprocessed data.

The patient consented to participation in a magnetic resonance imaging (MRI)-based research study. She underwent a perithrombolysis MRI scan with initial diffusion-weighted imaging confirming extensive acute ischemic stroke and no evidence of hemorrhage at that time (fig. 1a, c). Six minutes into the sequences, an increasing mass effect was noted on the reformatting images within the head of the right caudate and ventral putamen (fig. 1c). Alteplase was discontinued as this was thought to be secondary to intracerebral hemorrhage. At this time the patient was clinically stable, but she became obtunded over the following hour. Immediate CT confirmed extensive intraventricular hemorrhage with early hydrocephalus (fig. 1b). Following discussion with her family and in light of her clinical state, a decision was made to provide palliative care. The patient died 2 days later.

Reformatting of the imaging demonstrated the progression of the intracerebral bleed. In this video (online suppl. video 1; see www.karger.com/doi/10.1159/000355144 for all online suppl. material), a secondary hematoma can be seen shrinking as blood disgorges into the ventricles.

## Discussion

Hemorrhagic transformation is the most feared complication of intravenous thrombolysis and occurs in approximately 5% of patients treated with alteplase [2,3]. Predicting and defining those patients who will suffer this potentially life-threatening complication is challenging [4,5]. Here, we present a unique video of ventricular extension of a secondary hemorrhage in a patient receiving intravenous thrombolysis. The extent of the ischemic injury meant that the clinical manifestations of the hemorrhage did not occur until some time after the bleeding had extended into the ventricles.

## Acknowledgements and Sources of Funding

The research was supported by the National Institute for Health Research Oxford Biomedical Research Centre Programme, the Dunhill Medical Trust (grant No. OSRP1/1006) and the Centre of Excellence for Personalized Healthcare funded by the Wellcome Trust and Engineering and Physical Sciences Research Council under grant No. WT 088877/Z/09/Z. We wish to acknowledge the facilities provided by the Oxford Acute Vascular Imaging Centre.

## Disclosure Statement

Y.K.T. is funded by a Qualcomm Scholarship from Qualcomm Inc. The other authors declare no conflicts of interest.

## Supplementary Material

Suppl. video 1 Serial single slice images during a chemical exchange saturation transfer MRI sequence. Acquisition occurred over approximately 1 minute. A hematoma within the head of the right caudate and ventral putamen can be seen as it discharges blood into the right lateral ventricle. Following ventricular extension, the parenchymal hematoma decreases in volume. There is signal change first in the right and then in the left lateral ventricle as the blood displaces the cerebrospinal fluid.
